# Killer whale acoustic patterns respond to prey abundance and environmental variability around the Prince Edward Islands, Southern Ocean

**DOI:** 10.1098/rsos.230903

**Published:** 2024-01-03

**Authors:** Fannie W. Shabangu, Robyn Daniels, Rowan K. Jordaan, P. J. Nico de Bruyn, Marcel A. van den Berg, Tarron Lamont

**Affiliations:** ^1^ Fisheries Management Branch, Department of Forestry, Fisheries and the Environment, Foreshore, Cape Town, South Africa; ^2^ Mammal Research Institute, University of Pretoria, Private Bag X20, Hatfield, Pretoria 0028, South Africa; ^3^ Department of Oceanography, University of Cape Town, Cape Town, South Africa; ^4^ Nansen–Tutu Centre for Marine Environmental Research, University of Cape Town, Cape Town, South Africa; ^5^ Oceans and Coasts Research Branch, Department of Forestry, Fisheries and the Environment, Foreshore, Cape Town, South Africa; ^6^ Bayworld Centre for Research and Education, Cape Town, South Africa

**Keywords:** *Orcinus orca*, vocalizing behaviour, acoustic occurrence, sub-Antarctic region, prey, oceanographic variables

## Abstract

Killer whales are apex predators with temporally and spatially varying distributions throughout the world's oceans. Their ecology and behaviour are poorly understood in most regions due to limited research, often because of logistical challenges. Here, we used a passive acoustic monitoring device to investigate the seasonal acoustic occurrence and diel vocalizing behaviour of killer whales around the remote sub-Antarctic Prince Edward Islands (PEIs), Southern Ocean. Killer whales showed diel vocalizing patterns that varied seasonally in relation to their prey abundance and social activities. Killer whale calls were intermittently detected year-round with a high number of hours containing calls in October to December, and a secondary peak in February to May, corresponding to seal prey abundance. Random forest modelling identified wind speed as the primary predictor of the occurrence of killer whale calls (with a negative correlation) while sea surface height, chlorophyll-a and sea surface temperature were moderately important. We provide the first acoustic evidence that killer whale occurrence around the PEIs might coincide with variability in environmental conditions and prey abundance. Our results provide the first indication of diel vocalizing pattern of killer whales in the Southern Ocean. This knowledge is important for understanding killer whale ecology and adaptation to the changing oceans.

## 1. Introduction

Killer whales (*Orcinus orca*) are highly mobile apex predators with a worldwide distribution, inhabiting all major ocean basins [1–3]. Due to their high energetic requirements [4,5], killer whales exert top-down control on a diverse selection of prey, potentially affecting marine ecosystem structure and function at multiple trophic levels [4–8]. Several killer whale ecotypes (A, B, C and D) differing in morphology, genetics, diet, movement, foraging behaviour, acoustic repertoire and social structure exist within the Southern Ocean [2]. The distribution and occurrence of these killer whales are influenced by the occurrence of prey, suitable environmental conditions and sexual maturity [3,7,9,10]. Overall, there is a lack of information about their whereabouts in the open ocean [4,5] and the environmental factors responsible for their spatial variation are not well understood [9,11]. Studies dedicated to killer whale occurrence, distribution and abundance within the Southern Hemisphere are limited, with little research addressing the direct effects of the marine environment on their occurrence, behaviour and habitat preference [9,12]. This study therefore focused on investigating the influences of prey abundance and environmental conditions on the acoustic occurrence of killer whales around the Prince Edward Islands (PEIs).

**Figure 1.**
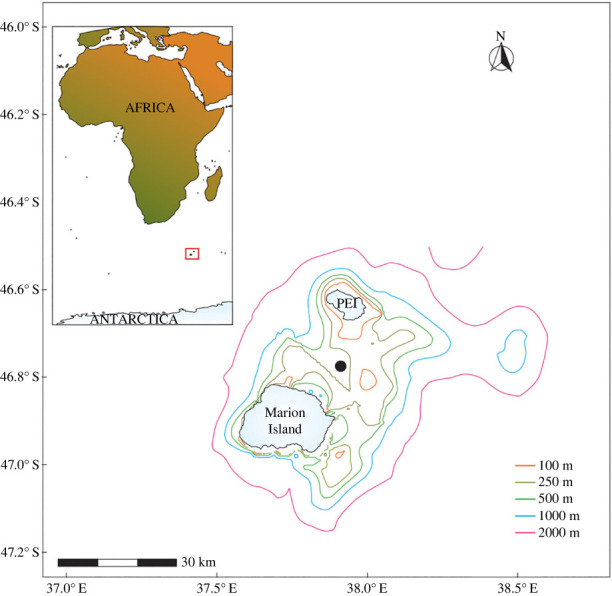
Map showing the location of Marion Island and Prince Edward Island (PEI) constituting the PEIs together with location of the oceanographic mooring containing the acoustic recorder (black circle). Bathymetry is indicated by colour-coded contour lines as shown in the key. Insert map shows the zoomed out position of the PEIs (small red box) relative to South Africa's mainland and Antarctica. Bathymetry data was obtained from the 2022 General Bathymetric Chart of the Oceans (GEBCO) Compilation Group (https://doi.org/10.5285/e0f0bb80-ab44-2739-e053-6c86abc0289c).

The sub-Antarctic PEIs archipelago is located at 46.9° S, 37.7° E in the Indian sector of the Southern Ocean and consists of the smaller Prince Edward Island (PEI; 44 km^2^) which lies approximately 19 km northeast of the larger Marion Island (290 km^2^; figure 1) [13]. The archipelago is 1800 km southeast of South Africa and 2300 km north of Antarctica (figure 1). The nearest land mass, the Crozet Achipelago, is 950 km due east on a similar latitude to the PEIs. The sub-Antarctic region, including the PEIs, is threatened by the rapid rise in temperatures and subsequent climate variability [14,15]. Environmental conditions around the PEIs are dynamic as this archipelago is situated in the direct pathway of the Antarctic Circumpolar Current (ACC) [16]. The ACC is the largest water transporting current in the ocean and is able to influence water masses and climate anomalies between all ocean basins [17]. The ACC consists of three major fronts—the sub-Antarctic front (SAF), Antarctic Polar front and the southern ACC front. These fronts are not singular features, but instead consist of a series of strong jets usually referred to as various branches [18,19]. Across each front are sharp gradients in water properties [17].

The ACC fronts tend to meander in the vicinity of the PEIs promoting mixing between warmer and colder waters. Substantial mesoscale eddy activity in the ocean and the passage of atmospheric low-pressure systems are also evident within the PEIs region [16]. The ACC and the prominent seafloor topography of the southwest Indian Ridge in the study region (figure 1) give rise to productively turbulent waters surrounding the PEIs [20–22]. Enhanced primary productivity promotes growth of killer whale prey resources, such as large masses of seals and seabirds which use the islands for breeding purposes [6,9,23]. Although the mechanisms are unclear, it has been suggested that these prey populations are sensitive to climate variability [9,24,25].

The PEIs killer whales, which are more genetically related to ecotype B [26], prey on southern elephant seals (*Mirounga leonina*), sub-Antarctic fur seals (*Arctocephalus tropicalis*), king penguins (*Aptenodytes patagonicus*), gentoo penguins (*Pygoscelis papua)*, macaroni penguins (*Eudyptes chrysolophus)* and Patagonian toothfish (*Dissostichus eleginoides*) [1,6,8,10]. Shore-based sightings determined that killer whales frequenting the inshore region of Marion Island rely on the high prey abundance [23,27,28], and nearby Patagonian toothfish fisheries (through depredation) for reproduction, social structure regulation and survival [10]. While the PEIs killer whale population has been observed here year-round, they have been reported to be most common between September and December [1,6,28].

Passive acoustic monitoring (PAM) is a useful tool to monitor spatio-temporal patterns of killer whale behaviour and occurrence, since PAM is a method by which animals are detected based on the sounds they emit [29]. This tool allows for data collection year-round and in an otherwise harsh environment where researchers are practically/logistically limited in their monitoring [30,31]. Nevertheless, PAM only detects vocally active cetaceans and does not detect silent animals. Killer whales are highly social marine mammals which rely on their vocal abilities to interact with other members of their travelling and foraging groups [32,33]. Stereotypical pulsed calls and tonal signals (i.e. whistles) are produced during social activities aiding group coordination, cohesion and recognition [34,35]. Echolocation clicks can be used as a mechanism for locating prey, and to navigate and interrogate surrounding environments [29,36].

Currently, the only Antarctic killer whale ecotypes that have been distinguished acoustically are ecotypes C and D [36–39] and this study gives us the opportunity to identify call types from another ecotype. The objective of this study was to describe the diel vocalizing pattern and determine the seasonal cycle of acoustic occurrences of killer whales and compare the latter with environmental conditions surrounding the PEIs.

## 2. Material and methods

### 2.1. Passive acoustic monitoring sampling approach

A SoundTrap ST500 STD autonomous recorder (Ocean Instruments NZ, New Zealand) deployed on an oceanographic mooring was used to collect the PAM data for this study. The oceanographic mooring consisted of an anchor, two acoustic releases, linking chains and a float which housed an acoustic Doppler current profiler (ADCP) and the acoustic recorder. Linking chains were interweaved with ropes between the chain links to reduce noise. This mooring system forms part of the South Atlantic Meridional Overturning Circulation (SAMOC) programme aimed at obtaining an optimal observation network to monitor the global overturning circulation in the South Atlantic Ocean and how it interacts with the Southern Ocean (https://www.aoml.noaa.gov/phod/SAMOC_international/). The oceanographic mooring was deployed approximately 10 km from the coast of Marion Island and 9 km from PEI (figure 1, table 1). Settings and sampling protocol for the autonomous recorder are detailed in table 1, where the acoustic recorder sampled the first 14 min of the second half of every hour of each day (e.g. from 06.30 to 06.44 and so on). We are considering the environment around the two PEIs as our acoustic study area, given that killer whale calls have been estimated to propagate up to 10 km by Richard *et al*. [36] around the neighbouring Crozet Archipelago, Southern Indian Ocean, which would enable calls from both islands to be detected by our acoustic recorder deployed between them (figure 1).

**Table 1.** Summary of deployment details and recording settings of the SoundTrap (ST) autonomous recorder used in this study. Hydrophone sensitivity is from factory calibrations of the HTI-96-MIN (High Tech Inc.) hydrophone.

Latitude (° S)	Longitude (° E)	Water depth (m)	ST depth (m)	Sampling rate (kHz)	Sampling protocol (min h^−1^)	Duty cycle (%)	Hydrophone sensitivity (dB re 1 V µPa^−1^)	Start recording date	Stop recording date
46.77	37.91	167	162	96	14	24	− 165	26/04/2021	06/05/2022

### 2.2. Acoustic occurrence determination

**Figure 2.**
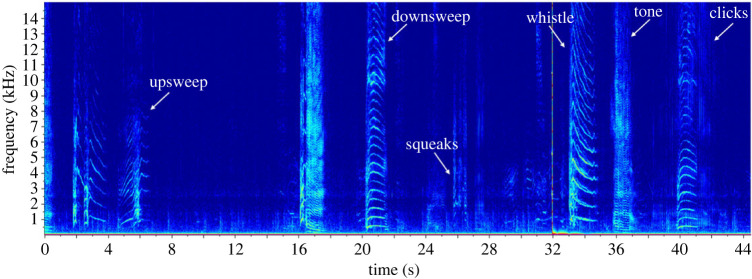
Different calls of killer whales detected around the PEIs. Harmonics were detected all the way up to the Nyquist frequency of recordings (i.e. 48 kHz). Spectrogram parameters were as follows: Hann window, frame size of 0.0394 s, a discrete Fourier transformation (DFT) size of 4096 samples and a 50% overlap.

Passive acoustic monitoring data were analysed aurally and visually using spectrograms (figure 2) in Raven Pro, version 1.6.3 [40] to identify and quantify the presence of killer whale calls. We used examples of killer whale calls provided in Schall & van Opzeeland [38] and Wellard *et al*. [39], and consulted two acoustic experts to confirm that detected calls were indeed killer whale calls. Since there is currently no classification catalogue of killer whale calls for the PEIs, we have grouped the detected calls into two general groups based on function of calls: echolocation clicks and social calls consisting of squeaks, downsweeps, upsweeps, whistles and tones (figure 2). Long-finned pilot whales (*Globicephala melas*) also produce clicks, pulsed calls and tonal signals similar in structure to those of killer whales [41], but these are rarely sighted (with two sightings since 2007; R.K.J. unpublished data) around the PEIs, hence we do not consider them as the source of these detected sounds. Humpback whales (*Megaptera novaeangliae*) are known to produce units within their songs [42], some of which are similar to killer whale social calls, and we differentiated these from killer whale social calls by their repetitive pattern and downsweeping from 1 kHz to low frequency (less than 500 Hz). Sperm whales (*Physeter macrocephalus*) also produce echolocation clicks [43], but these are usually found further offshore (greater than 500 m water depth) of the PEIs [44].

Firstly, spectrograms were evaluated at a maximum frequency of 15 kHz in 2 min timeframes to identify acoustic files with calls, and secondly at 15 kHz in 45 s timeframes to count calls. The spectrogram settings were as follows: Hann window, a frame size of 0.0394 s, a discrete Fourier transformation (DFT) size of 4096 samples and a 50% overlap. Killer whale call and click production rates were determined by manually counting the individual number of calls or clicks/click trains for each call category within each sampling session, and presented as calls per minute,

2.1 $$\mathrm{Call}\;\;\mathrm{or}\;\;\mathrm{click}\;\mathrm{rate}=\frac{\mathrm{Number}\;\mathrm{of}\;\mathrm{calls}\;\mathrm{or}\;\mathrm{clicks}}{\mathrm{Duration}\;\mathrm{of}\;\mathrm{sampling}\;\mathrm{session}\;},$$

where the duration of sampling session is 14 min.

Clicks within click trains and buzzes were considered as one bout of clicks and not counted to individual click level due to difficulty in manually counting and differentiating very closely spaced clicks that can range from hundreds to thousands of clicks within a minute [36,45]. Consequently, the estimated killer whale click rate of this study provides the lowest possible rate but nonetheless provides a good indication of click presence and foraging events.

Daily number of hours with killer whale calls were calculated for each call category as the total number of 14 min recordings containing at least one or more detections of each call category per day. Each 14 min recording represented the hour in which it was recorded; for example, 06.30–06.44 represented 06.00. These daily number of hours with killer whale calls indicated the acoustic occurrence of killer whales around the PEIs. Total acoustic effort (TAE; in hours) for the whole deployment period was calculated using equation (2.2),

2.2 $$\mathrm{TAE}=\overset{N}{\underset{i=1}{\sum }}TH_{i}\cdot ND_{i},$$

where *N* is the last day of the sampling month, *TH_i_* is total number of hours (calculated from 14 min sampling sessions as 14/60 = 0.23 h) for each day, and *ND_i_* is the total number of sampling days within each month. TAE for each month was calculated as *TH_i_* × *ND_i_*.

The austral seasonal cycle was used to describe our data: summer (December to February), autumn (March to May), winter (June to August) and spring (September to November).

### 2.3. Diel vocalization pattern

To explore diel vocalizing patterns of killer whales, call and click rates (from equation (2.1)) were used. Nautical daylight regime (sunrise, sunset and nautical twilight) for the PEIs were obtained for the oceanographic mooring location (table 1) using the ‘suncalc’ package [46] in R (version 4.2.3) [47]. Daytime was defined as the time between sunrise and sunset. Nautical dawn and dusk were defined as the time before sunrise and after sunset respectively when the sun was geometrically between 0° and 12° below the horizon. Night-time was defined as the time between dusk and dawn when the sun was more than 12° below the horizon. Statistical differences of call rates between daylight regimes for both call categories were tested through multiple pairwise t-test comparisons (with Bonferroni adjustment as a multiple testing correction) using the ‘rstatix’ package [48] in R.

### 2.4. Killer whale visual observations

To estimate the number of unique killer whales present around Marion Island, identification photographs were collected from Marion Island shore using various digital camera and lens combinations. These visual counts of unique killer whales in the area were later compared with acoustic detection to confirm that the recorded calls were produced by killer whales and to determine if the number of animals around the island influence the killer whale acoustic occurrence. Killer whales were photographed during opportunistic sightings (e.g. when observers were completing other fieldwork) or during dedicated observation sessions. Dedicated killer whale observation sessions were conducted by trained observers [49], during which they visually searched for killer whales for an uninterrupted 3–10 h period. When sighted, observers recorded the killer whale's group size, its movement direction and age/sex composition. Additionally, observers attempted to photograph the dorsal fin of each individual within the group while in photographic range. Observations were only conducted from the bigger island, Marion Island, occupied by observers, and not on the smaller PEI, as there are no observers based there. We expect similar occupancy between these two islands given their close proximity (approx. 19 km apart) to each other (and the PAM instrument being situated roughly halfway between them). Unique number of killer whales were estimated using a method described in electronic supplementary material, S1. Considering that only 29% of days during the sampling period contained killer whale observations, the other days without observations were filled with zeroes.

### 2.5. Southern elephant seal visual observations

All beaches on the eastern side of Marion Island (comprising all breeding beaches) were surveyed every 9 days during the southern elephant seal (SES) breeding season (August to November) and every 10 days outside of the breeding season. Surveys were used to estimate the abundance of SES (during 2021/2022), the main prey of killer whales around the PEIs [6,28]. From each survey cycle, the total number of seals present on the island during each survey period was obtained. The number of SES was not estimated from the smaller PEI since there are no researchers based on that island; however, the number of SES observed around the bigger Marion Island represents the majority of the SES in the PEIs population [50]. We filled days without SES observations (representing 29% of the sampling period), by grouping the data into 5-day groups and linked each date with the data from the nearest date before or after, this method is supported by the visual observations that SES linger within the same area for extended periods [51]. We did not consider other killer whale prey such as penguins and other seal species around the islands as their year-round abundance estimates are not available at high enough temporal resolution.

### 2.6. Environmental data

Ocean reanalysis and satellite-derived data of daily sea surface temperature (SST), daily sea surface height (SSH), daily chlorophyll-a and hourly (averaged to daily level to match the temporal scale of other variables) wind speed (table 2) were extracted and spatially averaged across a 2° (222 km latitude) × 2° (156 km longitude) area centred over the mooring system location (46°46.4′ S, 37°54.7′ E) deployed between the PEIs. We did not use the estimated maximum killer whale call detection range of 10 km [36] to extract environmental data as the spatial resolution of some of the satellite-derived variables, especially for SSH and wind speed, were only available at a 28 km spatial resolution (table 2) and thus too coarse to detect the local shelf dynamics, and might have been affected by land contamination [21]. Ocean reanalysis wind speed data extracted within 2° × 2° quadrants around the PEIs were previously found to have a good correlation with the *in situ* observations made from Marion Island [16]. Thus, the use of the 2° × 2° quadrants in this study captures the larger-scale variability within the study region and probably provides a robust indication of the environmental conditions experienced by killer whales around the PEIs. These data were processed in Python software, version 3.9, available at http://www.python.org.

**Table 2.** Summary of environmental variables derived from global environmental data repositories. The column ‘usage’ indicates the reasons why each environmental variable is used in this study. Group and product abbreviations are defined: CMEMS is Copernicus Marine Environment Monitoring Service, DUACS is Data Unification and Altimeter Combination System, ERA5 is fifth generation of European Centre for Medium-Range Weather Forecasts reanalyses, GlobColour is Global Ocean Colour for Carbon Cycle Research, and OSTIA is Operational Sea Surface Temperature and Sea Ice Analysis. GlobColour uses merged, gap-free data from multiple satellite sensors which include Sea-Viewing-Wide Field-of-View Sensor (SeaWiFS), the Medium Resolution Imaging Spectrometer (MERIS), the Moderate Resolution Imaging Spectroradiometer (MODIS) Aqua, the Visible Infrared Imaging Radiometer Suite (VIIRS NPP), and the Ocean and Land Colour Instrument (OLCI-S3A) sensors.

variable	unit	group: product	data repository link	spatial resolution	usage
chlorophyll-a (chl-a)	mg m^−3^	CMEMS: GlobColour	https://data.marine.copernicus.eu/product/OCEANCOLOUR_GLO_BGC_L4_MY_009_104/description	0.04° × 0.04° (4 × 3 km)	proxy for primary production and phytoplankton biomass
sea surface height (SSH)	m	CMEMS: DUACS	https://data.marine.copernicus.eu/product/SEALEVEL_GLO_PHY_L4_MY_008_047/description	0.25° × 0.25° (28 × 19 km)	locate the position of the ACC fronts around the PEIs and suitable habitat conditions for animals
sea surface temperature (SST)	°C	CMEMS: OSTIA	https://data.marine.copernicus.eu/product/SST_GLO_SST_L4_REP_OBSERVATIONS_010_011/	0.05° (6 km)	indicative of changes in physical oceanographic processes which affect primary productivity around the PEIs
wind speed^a^	m s^−1^	CMEMS: ERA5	https://cds.climate.copernicus.eu/cdsapp#!/dataset/reanalysis-era5-pressure-levels?tab=form	0.25° × 0.25° (28 × 19 km)	proxy of sea state conditions

^a^Absolute wind speed (*ws*) was calculated from meridional (*v*) and zonal (*u*) wind speeds as $ws=\sqrt{u^{2}+v^{2}}$.

### 2.7. Statistical data analyses

We determined if killer whale acoustic occurrence responded to variabilities in environmental conditions, prey abundance and number of killer whales sighted around the PEIs using the random forest (RF) model [52]. Partial effects and index of importance were used to indicate the probability of hourly killer whale acoustic occurrence. We used the RF model as it performs better than generalized additive models and generalized boosted regression trees models for assessing acoustic occurrence of other marine mammals [53,54]. Other distinctive and important features of RF models are that they have high prediction accuracy and non-parametric inferential properties whilst implicitly including variable interaction [52,55,56]. Predictor variables used were chlorophyll-a, SST, SSH, month, hour of day, wind speed, number of unique killer whales and total number of SES. Prior to fitting the RF models using electronic supplementary material, equation S1 (where individual RF models were fitted for each call category), a multi-collinearity test was conducted between predictor variables using generalized variance inflation factors (GVIFs) [57] implemented through the ‘car’ package [58] for the RF models to produce accurate indices of variable importance. No multi-collinearity was found between predictor variables as the highest GVIF was 2.14 indicating weak or no collinearity [57].

**Figure 3.**
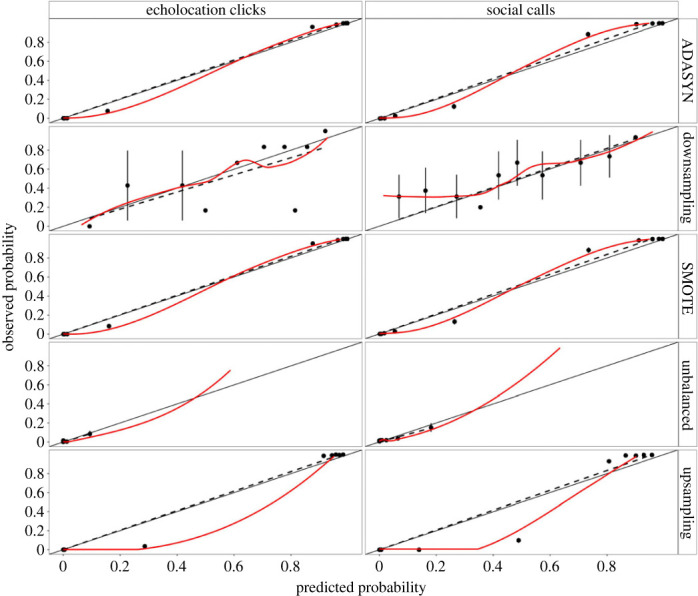
Calibration plots used to visually evaluate the RF model predictive performance for killer whale calls detected around the PEIs.

We tested four different methods of addressing the noticeable difference in class imbalance of acoustic detection of the two call categories (104 h with detections for echolocation clicks versus 254 h with detections for social calls): Synthetic Minority Over-sampling Technique (SMOTE) [59], ADAptive SYNthetic (ADASYN) [60], downsampling and upsampling [61]. Calibration plots indicated that downsampling, ADASYN and SMOTE methods had better probability distributions between predicted and observed data than the other two sample balancing methods (figure 3). However, the downsampling method was better calibrated than ADASYN and SMOTE as its data were spread across the observed proportions, its deviation always crossed the intercept, and its smoothed line was also close to the ideal diagonal line. Thus, the downsampling method was used to describe our data. Nonetheless, RF model results for ADASYN and SMOTE are provided in electronic supplementary material, figures S1 and S2, for comparison.

The RF models were tuned using 70% of the balanced data for training, and the remaining 30% was used for testing. The training dataset was further set up for fivefold cross-validation, which was in turn used for tuning the model. Predictive performances of all the RF models were then assessed on the test dataset, which was not used when tuning the model. For these classification RF models evaluating the influence of environmental conditions and prey abundance on the hourly acoustic occurrence of killer whales, the best tuning parameters were chosen as sets that maximize the area under the curve of the receiver operating characteristics. All feature importance values were scaled to the maximum for each model and presented as percentage. To allow easier interpretation of our RF model results, significance (*p*-value) of each feature importance value was tested using the permutation method of Altmann *et al*. [62]. The above RF modelling was performed using the ‘randomForest’ package [63] implemented through the ‘ranger’ package [64] as a faster method of fitting RF models in R.

## 3. Results

### 3.1. Passive acoustic monitoring effort

A TAE of 2100 h was made over 376 days (table 3). The TAE for the months of April 2021 and May 2022 was not at its maximum capacity, as the recorder only monitored for 26.83 h and 29.17 h during these months respectively, as a result of deployment and retrieval of the instrument.

**Table 3.** The TAE per month and for the whole sampling period.

year	month	hours recorded	number of days
2021	April	26.83	5
	May	173.6	31
	June	168	30
	July	173.6	31
	August	173.6	31
	September	168	30
	October	173.6	31
	November	168	30
	December	173.6	31
2022	January	173.6	31
	February	156.57	28
	March	173.6	31
	April	168	30
	May	29.17	6
	**Total**	**2100**	**376**

### 3.2. Acoustic occurrence and visual observations

**Figure 4.**
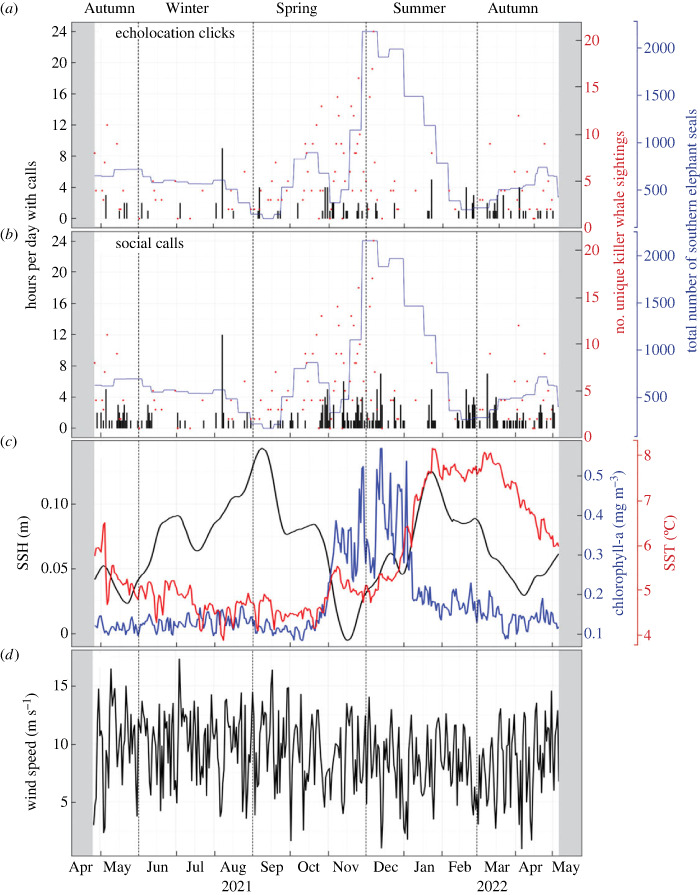
Time series data for hours per day with killer whale (*a*) echolocation clicks (bar plots) and (*b*) social calls (bar plots) around the PEIs, number of unique killer whale sightings (overlaid points) and the total number of sighted southern elephant seals (line plots) around Marion Island. The daily (*c*) SSH, chlorophyll-a, and SST; (*d*) wind speed around the PEIs over the study period. Grey shaded areas indicate dates prior to and after recorder deployment without PAM effort. The x-axes represent months of each year.

Observers conducted 175 dedicated killer whale visual observation sessions, totalling 954 h, at Marion Island from April 2021 to May 2022 (figure 4). During this time, 237 killer whale sightings were recorded with an average of 0.25 sightings per hour. An additional 314 killer whale sightings were opportunistically recorded during the same period. From these 551 killer whale sightings, 34 374 photographs were taken and a total of 36 unique killer whales were identified (representing 67% of the entire Marion Island population of 54 estimated by Jordaan *et al*. [49]). A mean of five individuals were observed per day when excluding days without sightings, while a maximum of 21 unique killer whales were observed on a single day in December 2021 (figure 4). In total, 40 SES surveys were conducted during the study period and SES were found throughout the year. An average of 693 seals were observed during each survey with a maximum of 2120 individuals observed during a single survey (figure 4).

Killer whale calls were detected intermittently throughout the year, and there were days when killer whales were sighted but not detected acoustically and vice versa (figure 4). A total of 3551 echolocation clicks and 10 581 social calls were enumerated from our year-round dataset. The highest number of hours with killer whale calls per day were observed in August 2021 for both call categories, where 9 h per day were the highest for echolocation clicks and 12 h per day were the highest for social calls (figure 4). Echolocation clicks and social calls depicted similar seasonal variability with peaks in austral spring (October and November) and early summer (December) and a secondary peak in late summer to autumn (February to May). Winter had the lowest occurrence for both call categories (figure 4). The seasonal cycle of the number of unique killer whales sighted displayed a similar pattern to the killer whale occurrence detected acoustically, peaking in spring (October and November) and summer (December). Three peaks in the total number of sighted SES were observed, one small peak in spring, another small peak in autumn, and the biggest peak in summer (figure 4). Our data suggest that there is a co-occurrence of SES sighted with killer whale calls. Moreover, these killer whale acoustic occurrences sometimes appear to change with environmental conditions (figure 4). For example, the low SST in August corresponded to a high number of hours with calls for both call categories.

### 3.3. Diel vocalization patterns

**Figure 5.**
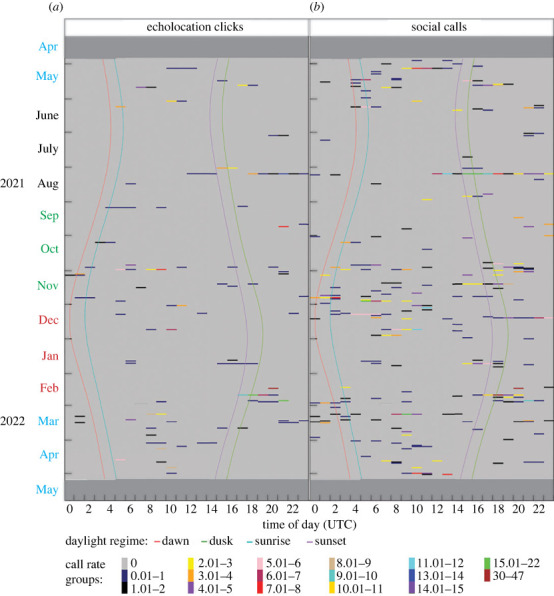
Hovmöller plots showing diel patterns of (*a*) echolocation click rates and (*b*) social call rates over different months and seasons. Time is indicated in UTC (Coordinated Universal Time). Dark grey shaded areas indicate periods without PAM effort. Daylight regimes are indicated by vertical lines described in the key, and call/click rate groups (grouped for better display) are described in the key. Months on the y-axes are colour coded according to season: blue indicates autumn, black indicates winter, green indicates spring, and red indicates summer.

Killer whale social call rates showed a strong diel vocalization pattern in winter, summer and autumn but a weak diel pattern in spring, while echolocation clicks only showed a strong diel pattern in summer and autumn with weak patterns in winter and spring (figure 5). Higher call rates were evident for both call categories during daytime compared with night-time in autumn (figure 5). A diel pattern was not visible for echolocation clicks in winter although slightly more clicks were detected at night on some days whereas social call rates increased from 11.00 to early morning (figure 5). Spring was characterized by high call rates during the day for both call categories and low call rates at night (figure 5). Both call categories followed the same diel pattern for summer with higher call rates during the day than at night (figure 5). The highest and second highest echolocation click rates were 35 and 22 clicks per minute respectively observed in summer (February) at night (20.00 and 21.00 respectively) (figure 5*a*). Summer (February) had the highest and second highest social call rates of 47 and 31 calls per minute respectively, both detected at night (at 18.00 and 19.00 respectively) (figure 5*b*). The average echolocation click rate for the whole study period was 0.028 ± 0.571 (standard deviation) clicks per minute, and 0.084 ± 0.928 calls per minute was the average call rate for social calls. Overall, day and night had significantly higher call rates than dawn but not dusk for both call categories (table 4). On the other hand, there was no significant difference between day and night for both call categories (table 4).

**Table 4.** Multiple pairwise comparisons of killer whale call rates at different daylight regimes. d.f. is degree of freedom, and *n* is sample size. Adjusted *p*-values are used for comparisons of significance, and unadjusted *p*-values are provided for information. Significance level threshold of *p* < 0.05 is used for test significance, and italic values indicate significance.

call category	variables compared	d.f.	unadjusted *p*-value	adjusted *p*-values
echolocation clicks	dawn : day	3660	*0.005*	*0.03*
	*n* = 1852 : *n* = 18 016			
	dawn : dusk	2490	0.072	0.432
	*n* = 1852 : *n* = 1888			
	dawn : night	12 390	*0.001*	*0.006*
	*n* = 1852 : *n* = 14 244			
	day : dusk	2155	0.578	1
	*n* = 18 016 : *n* = 1888			
	day : night	19 077	0.115	0.69
	*n* = 18 016 : *n* = 14 244			
	dusk : night	3639	0.667	1
	*n* = 1888 : *n* = 14 244			
social calls	dawn : day	3798	*0.002*	*0.012*
	*n* = 1852 : *n* = 18 016			
	dawn : dusk	2300	*0.027*	0.162
	*n* = 1852 : *n* = 1888			
	dawn : night	8880	*0.0002*	*0.001*
	*n* = 1852 : *n* = 14 244			
	day : dusk	2078	0.296	1
	*n* = 18 016 : *n* = 1888			
	day : night	22 502	0.125	0.75
	*n* = 18 016 : *n* = 14 244			
	dusk : night	2550	0.739	1
	*n* = 1888 : *n* = 14 244			

### 3.4. Environment around the Prince Edward Islands

Chlorophyll-a concentration was relatively low for most of the year, remaining between 0.08 and 0.2 mg m^−3^ during autumn and winter (figure 4*c*). There was a prominent increase in the chlorophyll-a concentration from late October to early January (late spring to mid-summer) with the maximum chlorophyll-a concentration of 0.57 mg m^−3^ observed in December (figure 4*c*). The SSH was mostly positive for the study period and two clear peaks were evident, one peak (0.14 m) in September (spring) and another peak (0.12 m) in January (summer) (figure 4*c*). The lowest SSH (−0.005 m) was observed in November (spring), and other comparatively low SSH values were observed for the days leading up to the end of May 2021 (autumn) as well as mid-April 2022 (autumn). There was northward movement of the southern branch of the sub-Antarctic Front (S-SAF) in May (autumn), November (late spring) and December 2021 (early summer), and the S-SAF was positioned south of the PEIs for the rest of the time (electronic supplementary material, figure S3). There was a clear seasonal cycle of SST during the year of our study, with the lowest SST of 4°C observed in early August (late winter), and fluctuations between 4 and 5°C for the remainder of August up until October (late winter until mid-spring) (figure 4*c*). SST increased from approximately 4°C to 5.5°C at the onset of November 2021 (late spring), and to 8.2°C by the end of January 2022 (mid-summer) and remained around 8°C until mid-March 2022 (autumn) when SST started to decrease through early May 2022 (late autumn). February and March (late summer and early autumn) had the highest average SST compared with other months (electronic supplementary material, figure S4a). Wind speed fluctuated daily and drastically between 5 and 15 m s^−1^, with high winds reaching a maximum of 17.3 m s^−1^ observed between April and September (mid-autumn to early spring) 2021 (figure 4*d*; electronic supplementary material, figure S4b). Generally, wind speed was lower than 14 m s^−1^ from November 2021 to April 2022 (late spring to mid-autumn) with the lowest value of 0.95 m s^−1^ observed in December (figure 4*d*). These results are detailed in electronic supplementary material, S5.

### 3.5. Predictors of acoustic occurrence

**Figure 6.**
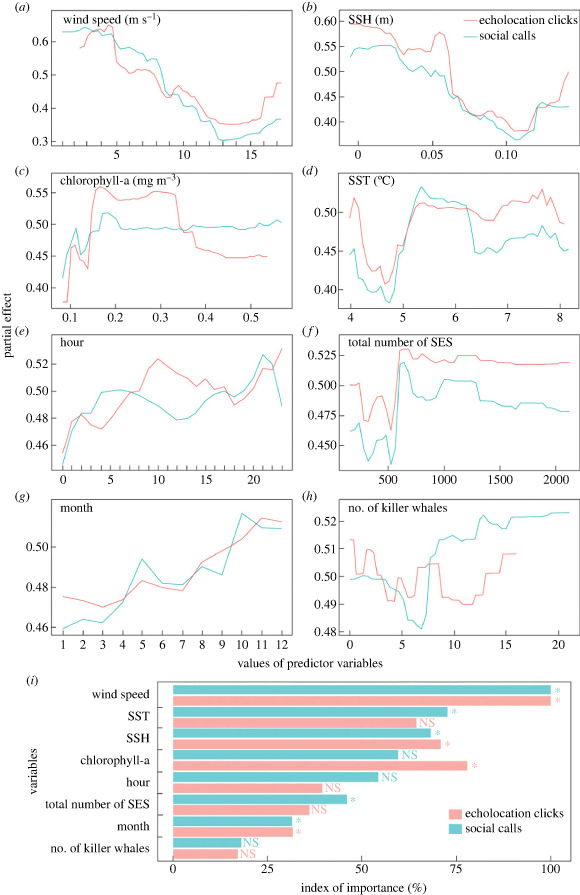
Random forest (RF) model (*a–h*) partial effects and (*i*) relative importance of predictor variables on the probability of the occurrence of echolocation clicks and social calls of killer whales based on the downsampling method. Y-axes scales are different between plots in (*a*) to (*h*). Asterisks (*) indicate significant importance (*p* < 0.05) and NS indicates non-significant importance (*p* > 0.05) based on Altmann *et al*. [62] method.

Probability of detecting both call categories was high at wind speeds below 8 m s^−1^ (figure 6*a*) and at low SSH below 0.07 m (figure 6*b*). The probability of detecting echolocation clicks increased as chlorophyll-a concentration increased from 0.15 to 0.35 mg m^−3^, while the probability of detecting social calls fluctuated with chlorophyll-a increase from 0.1 to 0.2 mg m^−3^ and then plateaued (figure 6*c*). Probability of detecting echolocation clicks was high at SST between 5.2 and 6.5°C, and 7 and 8°C, whereas social calls were most likely to be detected at SST between 5.2 and 6.2°C (figure 6*d*). Echolocation clicks were more likely to occur between 07.00 and 17.00 and from 21.00 to 23.00, while social calls were more likely to occur between 03.00 and 11.00, and between 14.00 and 23.00 (figure 6*e*). The probability of detecting both call categories was high when the total number of SES sighted was 600 but decreased as the number of SES increased at a faster rate for social calls than for echolocation clicks (figure 6*f*). Probability of detecting echolocation clicks was high in November and December, whereas social calls were more likely to be detected from October to December (figure 6*g*). There was a secondary high probability of detecting both call categories in May. Unique number of killer whales sighted had fluctuating effect on echolocation clicks, whereas the probability of social calls was high when unique number of killer whales sighted was above eight (figure 6*h*).

Wind speed was the most important predictor of the occurrence of echolocation clicks and social calls (figure 6*i*). Chlorophyll-a, SSH and SST were moderately important predictors of the occurrence of echolocation clicks, whereas SST, SSH, chlorophyll-a and hour of day were moderately important predictors of the occurrence of social calls. Hour of day, total number of SES sighted, month and unique number of killer whales sighted were the least important predictors of the occurrence of echolocation clicks, while total number of SES sighted, month and unique number of killer whales sighted were the least important predictors of the occurrence of social calls (figure 6*i*). Wind speed, SSH, chlorophyll-a and month were informative variables to predict the occurrence of echolocation clicks as they were significantly important predictor variables, meanwhile wind speed, SSH, SST, number of SES sighted and month were informative variables for predicting the occurrence of social calls (figure 6*i*). SST, hour of day, number of sighted SES and number of killer whales sighted were not informative variables for predicting the occurrence of echolocation clicks as they were not significantly important predictor variables. Chlorophyll-a, hour of day and month were not informative variables for predicting the occurrence of social calls as they were not significantly important predictor variables (figure 6*i*).

## 4. Discussion

Using echolocation clicks and social calls, we demonstrated for the first time the seasonal acoustic occurrence of killer whales around sub-Antarctic Islands in the Indian Ocean side of the Southern Ocean. This study documents the first diel acoustic behaviour of killer whales in the Southern Ocean and shows that they were more vocally active during the day in most seasons.

### 4.1. Diel acoustic behaviour

Diel vocalizing behaviour of killer whales indicated a strong pattern for most seasons, a suggestion of adaptive and diverse vocalizing patterns probably linked to their dynamic foraging behaviour, social structure and activities [1,7,10,27,32,33]. These killer whales produced equally high rates of echolocation clicks during daytime and night-time in autumn and spring, but produced higher click rates at night in summer, which indicates that they might have been hunting around the PEIs when the SES abundance was high [1,5]. A higher echolocation click rate was observed at night in winter when SES abundance was relatively low, another adaptive behaviour of this species in response to prey abundance [10,28]. Summer and winter were characterized by high social call rates before and after sunset respectively, perhaps reflecting that important social activity could be linked to group movement and/or hunting strategies of pods at that time of day [1,33,65].

Social call rates were high during the day in correspondence with echolocation clicks in autumn as killer whales were probably required to share important information during social activity, navigating or maintaining cohesion with their group mates while hunting [32,33]. Interestingly, social calls exhibited a strong diel vocalizing pattern towards the end of spring (just like echolocation clicks) with high call rates during the day likely to maintain contact acoustically as the number of killer whales sighted increased. This study's estimated call rates (maximum of 47 calls per minute for social calls) are considerably higher than those reported for Icelandic herring-eating killer whales [65], but the average social calls are comparable to average whistle call rates in Iceland [65]. The maximum echolocation click rate of 36 clicks per minute from this study is significantly lower than hundreds to thousands of clicks per minute observed for fish-eating resident and offshore killer whale ecotypes but comparable to mammal-eating transient killer whale click rate in the Northeastern Pacific [45]. No comparable estimates were found in literature for the Southern Hemisphere.

The increased vocalizing behaviour at sunrise to sunset around the PEIs, with a peak at mid-morning in spring, summer and autumn, corresponds to the increased foraging behaviour during those times seen for tagged North Atlantic killer whales off northern Norway [66]. High call rates at night for both call categories in some seasons are comparable to the Icelandic herring-eating killer whales that fed and vocalized more at night [65]. Satellite tracking results from Reisinger *et al*. [7] showed that killer whales around the PEIs dived to deeper depths during the day than at night, corroborating our results and perhaps indicating that acoustic communication is required more during the day to communicate in the deep and dark environment. Shallow dives at night around the PEIs were associated with hunting for seals and penguins inshore, local travel and socializing [7], which corresponds to those high call rates for both call categories in spring and summer.

The low call rates at night and early morning for both call categories in winter suggest that the PEIs killer whales might not use acoustics for communication when underwater light levels are reduced or could have been resting on the sea surface or underwater at those times [1]. Alternatively, they could be hunting silently during those times to reduce chances of acoustic detection by prey as has been observed for the mammal-eating killer whales in the Northeastern Pacific [67]. Lastly, these reduced call rates at night could indicate that animals were outside the detection range of the acoustic recorder. This study documents the first diel behaviour and acoustic activity of killer whales around the PEIs and the Southern Ocean (but see [1,68]). We provide the first estimates of call rates for killer whales in the Southern Ocean. Our estimated call rates provide the first indication of how frequently these killer whales vocalize and provides a useful reference for future studies in the Southern Ocean.

### 4.2. Killer whales, seals and the environment

The high number of killer whales sighted in October to December (mid-spring to early summer) corresponded to high detection of calls reflecting that sighted killer whales used the islands as the destination and not in transit, as travelling killer whales are generally silent [69]. These animals probably use the PEIs as their preferred feeding habitat given the high abundance of prey, especially SES at this time, linked to increasing chlorophyll-a concentration [6,7,9,22,28,70]. Nonetheless, the absence or low number of killer whale calls when animals were sighted and vice versa indicates that they might have been travelling via the islands or hunting silently at certain times of the year to avoid detection by their main prey, SES, who are probably acoustically vigilant underwater just like their Northern Hemisphere conspecific, northern elephant seals (*Mirounga angustirostris*) [71]. Alternatively, negative correlation might be due to limited (where 29% of the time contained data) sightings of killer whales for the whole study period. Increased acoustic presence of killer whales observed in October to December corresponds to the increased number of SES during the haul-out period in their breeding season (August to November) [72] and moulting season (December to March) [51] around the PEIs. Since SES are loyal to their natal sites [51], their presence and return to the PEIs can be predictable, making them a reliable and abundant prey of killer whales [6,7,10,28].

Killer whales showed diverse responses to environmental variability and prey abundance depending on the call category. The influence of chlorophyll-a on occurrence of echolocation clicks according to the RF model was high for values from 0.15 to 0.35 mg m^−3^, which overlapped with high abundance of SES when the biological productivity of the area was elevated [22,70]. On the contrary, the RF model partial effects of chlorophyll-a on social calls showed no clear response as this call category is not used for hunting. The RF model identified daytime (07.00 to 17.00) and midnight hours (21.00 to 23.00) to have the highest influence on the occurrence of echolocation clicks, suggesting that the PEIs killer whales hunted mostly during the day but with some limited hunting at night accompanied by vocalization. Condy *et al*. [1] similarly observed high killer whale feeding activities at dawn and dusk around Marion Island. Social calls were most likely to occur in the early morning to midday (03.00 to 11.00) and afternoon to midnight (14.00 to 23.00), indicating the critical socializing periods of this species.

RF model results indicated that the occurrence of echolocation clicks fluctuated with the increase in number of killer whales sighted around Marion Island, supporting the notion that the PEIs killer whales have complex foraging strategies driven by their adaptive social structure [10,22,28]. The number of killer whales (when eight or more animals were in the vicinity of Marion Island) resulted in an increase in the occurrence of social calls, which validates the dependence of large groups on acoustics for communication [35]. October to December had the highest influence on the occurrence of both call categories, and these months coincided with high SES abundance which supports the importance of prey availability on driving the social structure, behaviour and foraging of the species [7,10,28]. The secondary peak of killer whale call occurrence in May corresponds to the autumn peak of SES (figure 4) and when more sub-Antarctic fur seal pups start to spend more time in the shallows in that season [25].

High partial effects of SSHs below 0.07 m on both call categories suggests that those values provide suitable year-round marine habitats for killer whales [7,9] and their prey [24,25,73]. Thus, the Southern Ocean fronts are important foraging grounds for killer whale prey resources [16,24,25,73], and variability in the positions of these fronts can be expected to influence the distribution of killer whales and their prey [9]. SST between 5.2 and 6.5°C was identified by the RF model to have the highest partial effect on the occurrence of echolocation clicks and social calls. Reisinger *et al*. [7] similarly found SST to have a high effect on the movement, diving and hunting behaviour of killer whales around the PEIs. Remarkably, our results also showed that SST between 7 and 8°C had a high effect on echolocation click occurrence, which is sensible since these SSTs were observed in summer and autumn when SES were most abundant [6]. Furthermore, the occurrence of killer whales in these varying SSTs provides another indication of the great adaptability of this species to the high environmental variability of this region given that those measurements were comparable to the long-term SST climatology ([14,16]; electronic supplementary material, figure S4a).

Probabilities of occurrence of both call categories were high at low wind speeds below 8 m s^−1^ as these low wind speeds correspond to the seasonal cycle of wind (which is lower in spring/summer and higher in winter) reported by Toolsee *et al*. [74] and our calculated long-term wind speed climatology (electronic supplementary material, figure S4b). These low wind speeds also matched the high numbers of killer whales observed from October to December [28]. High wind speeds are associated with high underwater noise that could have masked killer whale calls in winter as wind-induced noise dominates underwater noise levels above 500 Hz [75], and wind-induced air bubbles on the sea surface could have attenuated some of the acoustic energy of the calls hitting the sea surface [76,77]. The observed secondary high partial effect of high wind speed could be because the wind pattern for mid-2021 to mid-2022 was somewhat different to the climatological seasonal cycle (electronic supplementary material, figure S4b) and coincided with killer whale occurrence. This response of killer whales to high wind speed also indicates an adaptation of these animals to the soundscape of the PEIs that might be dominated and driven by wind-induced noise since there is little to no vessel traffic. Wind speeds during autumn were higher than usual but corresponded with high killer whale acoustic occurrence, which probably caused the increased RF model response at wind speeds above 14 m s^−1^.

Wind speed was the most important predictor of the occurrence of both echolocation clicks and social calls as their occurrence in October to December coincided with low wind speed known for this area ([16]; electronic supplementary material, figure S4b). In addition, low wind speed would have improved the acoustic detectability of killer whale calls by our recorder and between individuals given the low ambient noise levels and increased the communication space between conspecifics at those wind speeds [43,75] and provided suitable habitat [9]. A combination of SSH, chlorophyll-a and SST were the moderately important predictors of the occurrence of echolocation clicks and social calls, a reflection of the importance of these variables at driving the ecology and foraging behaviour of killer whales in this region. Appropriately, these variables were previously found to be important for the movement, diving, foraging and social structure of killer whales around the PEIs [7,9]. Furthermore, these predictors are indicators of suitable environment for both killer whales and their prey [7,25].

Hour of day was moderately important for predicting the occurrence of social calls, suggesting that these calls were produced predictably at certain times of the day, whereas clicks were random throughout the day, thus hour was their least important predictor of this call category. Month and unique number of killer whales sighted were the least important predictors due to less dependence of killer whales on these variables compared with other variables, but these were nonetheless informative for predicting the occurrence of the hunting strategies of the species. Total number of SES sighted, month and number of unique killer whales sighted were the least important predictors of the occurrence of social calls because killer whales seemed to communicate and socialize with less effect from these variables. All significantly important predictor variables can be informatively used to predict the acoustic occurrence of killer whales, whereas all non-significantly important predictor variables signified that those could not be informatively used to predict call occurrence. However, it is worth noting that non-significance of predictor variables does not imply that those variables do not influence the acoustic occurrence of killer whales but simply imply that the random variation of those variables was too small for RF model to find a significant effect, although this does not mean that such effect cannot exist.

### 4.3. Conclusions

This study shows that there is probably a strong relationship between killer whale acoustic occurrence and environmental conditions (such as wind speed, SST and time of day) and their prey, southern elephant seals. Killer whales showed adaptive behaviours to the variability of the environment and prey abundance by being present year-round in this highly variable region. Daily ocean reanalysis and satellite-derived environmental conditions for the study period were generally similar to the long-term climatological variability of the region. We document the first diel acoustic behaviour of killer whales around the PEIs and the Southern Hemisphere, with an indication that killer whales are more vocally active during the day for most seasons. Moreover, we provide the first determination of seasonal acoustic occurrence of this killer whale population in the Southern Ocean. This study demonstrates that PAM can be used to study occurrence and behaviour of delphinids in the remote and isolated areas such as the PEIs under challenging and harsh environmental conditions.

## Data Availability

Data are provided as electronic supplementary material [78], in the form of acoustic .wav file of all exemplar calls illustrated by the spectrogram in the manuscript figure, and an MS Excel Spreadsheet file with killer whale call occurrence, seal count, killer whale sighting, daylight regimes and environmental data [79]. Links for downloading environmental data are provided in table 2.
